# Influences of bioplastic polylactic acid on near-infrared-based sorting of conventional plastic

**DOI:** 10.1177/0734242X211003969

**Published:** 2021-04-09

**Authors:** Xiaozheng Chen, Nils Kroell, Ke Li, Alexander Feil, Thomas Pretz

**Affiliations:** Department of Processing and Recycling, RWTH Aachen University, Aachen, Germany

**Keywords:** Bioplastic, near-infrared spectroscopy, polylactic acid, recycling, degradation, characterization

## Abstract

Bioplastics are developed to replace oil-derived plastics due to the high consumption of oil and related environmental impacts of oil-derived plastics. It was predicted that bioplastics can potentially replace 94% of conventional plastic production. With their increasing market share, more bioplastics will end in conventional post-consumer plastic waste streams. Although part of bioplastics is biodegradable and could be biologically decomposed, mechanical recycling achieves higher ecological benefits mainly because of its low pollution risk and the reduction in requirement for virgin feedstock. In this study, the classification of lightweight packaging waste with inflow of bioplastics, more specifically polylactic acid (PLA), was analysed with near-infrared spectroscopy to evaluate the influence of bioplastics on sorting processes of conventional plastics. Besides which, the sortability of PLA was determined through investigating the physical and the spectroscopic characteristics of both non-degraded and degraded PLA. The results show that the classification of all the materials was possible with a pixel-based accuracy of higher than 97.4% and PLA does not influence the sorting process of conventional plastics regarding detection and classification. Furthermore, the sorting of PLA from post-consumer waste is possible, which makes further recycling theoretically achievable.

## Introduction

Biobased and biodegradable plastics are produced and applied in different areas as a consequence of the high consumption of non-renewable crude oil for the production of fossil-based plastics and the generated environmental problems (European Bioplastics, 2018), for example, ocean plastic pollution (Jambeck et al., 2015). According to the study of Shen et al. (2009), bioplastics have the potential to replace 94% of oil-derived conventional plastics. One of the most widely used bioplastics is polylactic acid (PLA), which is derived from fermented plant starch and is completely biodegradable (Emadian et al., 2017). The market share of PLA was predicted to rise in the next decades and could reach up to 16.2% of plastic production (Shen et al. 2009).

With the increasing market share of bioplastics, the processing of end-of-life bioplastics needs to be considered (Rujnić-Sokele and Pilipović, 2017). Although part of biobased plastics is biodegradable, degradation is not the only or the most optimal treatment method for biodegradable plastic waste. According to life cycle analysis, mechanical recycling of plastics achieves the highest environmental benefits because of its relatively simple process, low pollution risk and the reduction in requirement for virgin feedstock (Dilkes-Hoffman et al., 2019). Most of the biobased and biodegradable plastics can be processed with conventional waste management options and have thus the potential to be reproduced to new products (Colwill et al., 2010, Dilkes-Hoffman et al., 2019). A closed-loop recycling system for specific post-industrial bioplastics, for example, PLA, has been developed by RE|PLA Cycle GmbH (Recyclingmagazin, 2012).

Post-consumer bioplastics that are disposed of will end in the waste flow together with conventional plastics, for example, in lightweight packaging (LWP) waste in Germany. However, the existence of PLA in recyclates of other materials can cause a deterioration of properties (Rujnić-Sokele and Pilipović, 2017), for example, the maximum proportion of PLA which does not influence the material properties is for polypropylene (PP) 3 wt%, for high-density-polyethylene (HDPE) 2 wt%, for polyethylene terephthalate (PET) 0.1 wt% and for polystyrene (PS) 10 wt% (Cornell, 2007; European Bioplastics, 2015; Hiebel et al., 2017; Niaounakis, 2013). Moreover, sorting of PLA enables further mechanical recycling, which brings benefits to the environment and a reduction of exploitation of primary resources (Dilkes-Hoffman et al., 2019).

To determine influences on detection and recycling of bioplastics in conventional post-consumer plastic waste and ensure an accurate sorting, LWP waste with PLA was analysed and sorted with near-infrared (NIR) spectroscopy – the state-of-the-art technology in plastic sorting (Feil and Pretz, 2020). The aim of this research is to determine the possibility of classifying PLA and other main conventional plastics. For this purpose, the PLA samples were degraded under laboratory conditions, and the samples with different degradation levels were recorded and analysed with NIR spectroscopy. Furthermore, to ensure the sorted and recycled quality of PLA products, the possibility to distinguish non-degraded and degraded PLA samples was investigated.

## Material and methods

In Germany, post-consumer plastic packaging waste is collected together as LWP waste, which is chosen for the simulation of the sorting process of conventional plastics and PLA in this investigation. The degradation of PLA samples and the detection as well as the classification analysis were carried out in laboratory scale.

### Sample selection and collection

**Figure 1.**
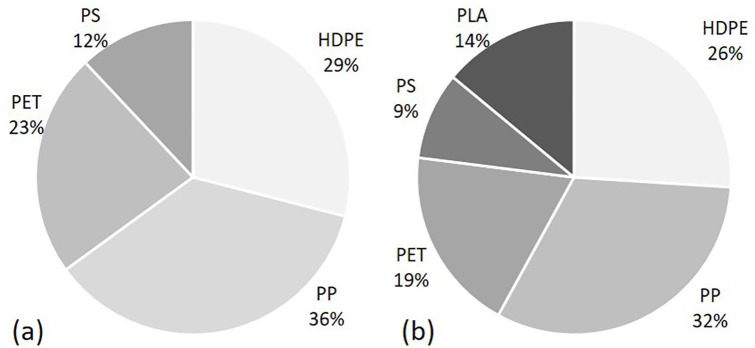
(a) Approximate proportion of polypropylene, high-density-polyethylene, polyethylene terephthalate and polystyrene in lightweight packaging waste (Interessengemeinschaft der Thermischen Abfallbehandlungsanlagen in Deutschland, 2015); and (b) the composition of applied materials.

As reported by Interessengemeinschaft der Thermischen Abfallbehandlungsanlagen in Deutschland [German Association of Waste-to-Energy Plants] (2015), the four dominant types of LWP are PP, HDPE, PET and PS, which account for 70 wt% to 90 wt% of the total amount of the three-dimensional fraction in LWP waste. In most post-consumer waste processing plants, only these four types of plastics are sorted (Interessengemeinschaft der Thermischen Abfallbehandlungsanlagen in Deutschland, 2015). For this reason, these four materials were collected and applied for the simulation and analysis. Figure 1(a) shows an approximate proportion of each material in the post-consumer LWP waste (Interessengemeinschaft der Thermischen Abfallbe-handlungsanlagen in Deutschland, 2015). According to the study of Shen et al. (2009), PLA has the potential to replace 10 wt% of PP, 10 wt% of HDPE, 20 wt% of PET and 10 wt% of PS. Based on this, the material composition for the sorting analysis with a total mass of 2.5 kg was defined (see Figure 1(b)).

The PLA samples were transparent disposable cups, which is the most common application of PLA in the household area and ends most likely in LWP (Shen et al., 2009). The samples made of conventional plastics were collected from German LWP waste. PLA cups were provided by the company Huhtamaki Foodservice (Alf/Mosel, Germany).

### Degradation of PLA

The degradation of PLA takes place under humid conditions with a rising temperature (Shen et al., 2009). Through laboratory tests, it was proven that with a temperature of higher than 58°C and a non-neutral, especially alkaline environment, the degradation process of PLA is much faster (Adam et al., 2016; Piemonte and Gironi, 2013; Scaffaro et al., 2019; Shen et al., 2009). In this research, the samples made of PLA were degraded in an alkaline environment with a pH-value of 10 and a temperature of about 65°C to accelerate the degradation process. After 1, 3, 5, 7, 14 and 21 days, the samples were dried and analysed with NIR spectroscopy.

### Spectra analysis

**Figure 2.**
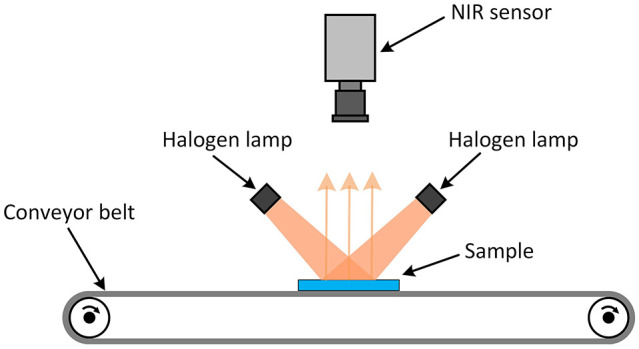
Near-infrared sensor and halogen lamps arrangement.

The NIR spectra of the samples were captured with a Helios-G2-320 NIR sensor from EVK DI Kerschhaggl GmbH (Raaba, Austria) in a spectral range of approximately 930 nm to 1700 nm. The spectral resolution is 3.1 nm/pixel, and the spatial resolution is 0.8 mm/pixel. Two halogen lamps with a power of 300 W each were used as emitters. The reflection of the radiation from the surface is captured by the NIR sensor, as shown in Figure 2.

The classification of plastics with NIR spectroscopy is owing to their specific NIR-active chemical structures and thus a characteristic spectrum in NIR wavelength area is obtained (Masoumi et al., 2012). The difference in spectra is represented by changing of the intensities of the reflected radiation. Consequently, the first derivative of the reflected radiation was used for determining the difference and for the classification. The classification was conducted with a partial least squares (PLS) discriminant analysis algorithm, and a component number of 10 was chosen. The implementation was based on the function “PLSRegression” from Scikit-learn (Pedregosa et al., 2011). To precisely determine the proportion of misclassification, the spectral data were pixel-based classified.

## Results and discussion

### Classification of conventional plastics and non-degraded PLA

**Figure 3.**
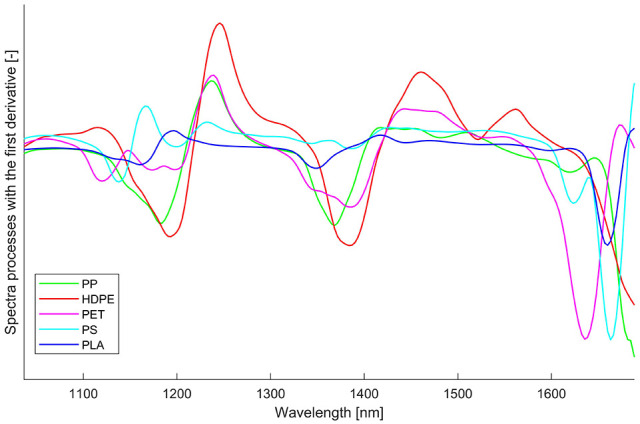
The mean spectra of polypropylene, high-density-polyethylene, polyethylene terephthalate, polystyrene and polylactic acid, processed with first derivative.

The spectra of the conventional plastics and non-degraded PLA were firstly compared to each other, and the samples were classified to determine the sortability. Figure 3 shows the mean spectra processed with first derivation of the conventional plastics and PLA.

From Figure 3 it is seen that different kinds of plastics have their own particular spectra. The difference between each material is sufficiently significant for a proper classification of the particles, and the classification accuracy of all the pixels was higher than 99%. From the detection and classification point of view, the maximum acceptable mass percentages of PLA in other material fractions were not reached, which means that the inflow of PLA in LWP would not influence the sorting of conventional plastics. In addition, the sorting of PLA from post-consumer waste is theoretically possible, which is the precondition for further processing and recycling.

### Influences of PLA degradation

**Figure 4.**
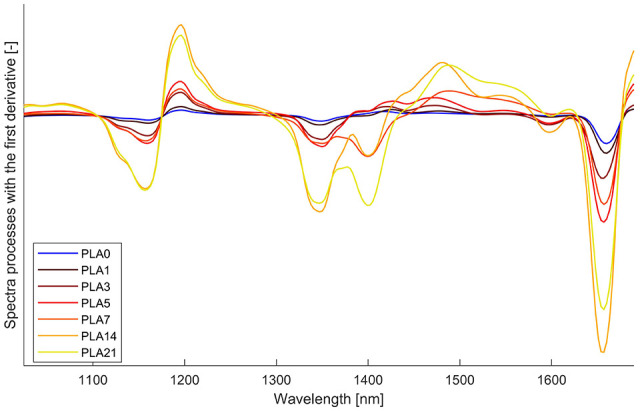
Spectra of polylactic acid (PLA) in different degradation levels (PLA*<*n*>*: PLA samples after *n* days degradation).

The samples with different degradation levels were analysed both in physical aspects and with NIR spectroscopy. With the degradation time extended, the colour of the samples has changed from transparent gradually to opalescent. In addition, the samples were becoming more and more brittle and after 14 days, they could no longer keep their original form but were present in small pieces. Samples after 7 days of degradation were too brittle so that a mechanical processing of these samples would be infeasible. The mean spectra of PLA in different degradation levels processed with first derivation are shown in Figure 4.

As shown in Figure 4, the general form of the spectra of the first 7 days remains almost unchanged. However, there are still slight differences in the spectra: the longer the degradation is, the higher is the intensity of peaks. One possible reason is that the samples are gradually less transparent and thus absorb and reflect more radiation in specific wavelength area. In this way, the intensity of the peaks increases. After 7 days, new peaks occurred in the wavelength area from about 1350 nm to 1450 nm, and with time extended, the intensity of the peaks increased. The reason could be the release of new chemical bonds, which absorb energy of radiation in this wavelength area.

Based on this observation, it was investigated if PLA samples with different degradation levels can be differentiated from each other by the PLS classification algorithm. As the samples after 7 days were not able to be mechanically processed (too brittle), the classification between “mechanical treatment possible” (⩾5 days degradation) and “mechanical treatment not possible” (⩾7 days degradation) was determined. With the used PLS algorithm, a classification accuracy of higher than 96.5% was achieved. This is owing mainly to the appearance of new characteristic peaks. Consequently, a sensor-based quality control of PLA is theoretically possible, as new peaks occur when new kinds of materials or chemical bonds are released.

After degradation, the samples were mixed with other kinds of plastics and analysed again with NIR spectroscopy. Although the spectra of PLA after degradation changed slightly because of new peaks, the detection and classification of all the materials were successful with a classification accuracy of 97.4%, as the spectra of degraded PLA keep the characteristic peaks of non-degraded ones and the variation is acceptable to be classified as the same material.

## Conclusion

Through analysis and classification of the main conventional plastics in LWP (PP, HDPE, PET and PS) and PLA with NIR spectroscopy, it was determined that from a detection and classification point of view, the sensor-based sorting of conventional plastics and bioplastics is possible. Both non-degraded and degraded PLA can be clearly differentiated from conventional plastics (PP, HDPE, PET and PS) by NIR spectroscopy. In order to avoid an inflow of bioplastics in the product fraction of conventional plastics, sorting recipes of existing sensor-based sorting equipment should be adjusted to supress a false ejection of bioplastics. Furthermore, additional sorting stages may be required to separate bioplastics to generate a pure bioplastic fraction for further processing. In addition, PLA should be sorted out shortly after collection to keep the original form and physical characteristics, and to avoid an unwanted degradation. Our findings that non-degraded and degraded PLA can be differentiated by NIR spectroscopy may be used to sort the bioplastics’ fractions into one fraction for mechanical recycling and another for further degradation (composting). For a more accurate determination of degradation levels, samples in different colour, transparency and more degradation levels should be investigated in further research.

## References

[bibr1-0734242X211003969] Adam F Sushant A Rakesh G (2016) Retarding hydrolytic degradation of polylactic acid: Effect of induced crystallinity and graphene addition. Journal of Applied Polymer 133: 44166.

[bibr2-0734242X211003969] Colwill JA Wright EI Clegg AJ, et al. (2010) Opportunities for bio-polymer resource conservation through closed loop recycling. In: Proceedings of the society of plastics engineers - Global plastics environmental conference 2010 (GPEC 2010), Orlando, USA, 8–10 March 2010, 7p. Society of Plastics Engineers Plastics Environmental Division.

[bibr3-0734242X211003969] Cornell DD (2007) Biopolymers in the existing postconsumer plastics recycling stream. Journal of Polymers and the Environment 15: 295–299

[bibr4-0734242X211003969] Dilkes-Hoffman LS Pratt S Lant PA, et al. (2019) The role of biodegradable plastic in solving plastic solid waste accumulation. In: Plastics to Energy: Fuel, Chemicals, and Sustainability Implications. Plastics Design Library. Amsterdam: William Andrew Publishing, 469–505. Available at: https://www.sciencedirect.com/science/article/pii/B9780128131404000194

[bibr5-0734242X211003969] Emadian S Onay T Demirel B (2017) Biodegradation of bioplastics in natural environments. Waste Management 59: 526–536. 27742230 10.1016/j.wasman.2016.10.006

[bibr6-0734242X211003969] European Bioplastics (2015) The behaviour of bioplastic films in mechanical recycling streams. Available at: https://docs.european-bioplas-tics.org/publications/bp/EUBP_BP_Bioplastic_films_in_mechanical_recycling_streams.pdf (accessed 15 June 2019).

[bibr7-0734242X211003969] European Bioplastics (2018) Bioplastics Market Data 2018. Berlin: European Bio-plastics. Available at: https://www.european-bioplastics.org/market/ (accessed 27 December 27).

[bibr8-0734242X211003969] Feil A Pretz T (2020) Mechanical recycling of packaging waste. In: Lechter M (ed.) Plastic Waste and Recycling: Environmental Impact, Societal Issues, Prevention, and Solutions. Amsterdam: Elsevier, 283–319.

[bibr9-0734242X211003969] Hiebel M Maga D Kabasci S, et al. (2017) PLA-Abfälle im Abfallstrom. Available at: https://news.fnr.de/fileadmin/allgemein/pdf/Pressemitteilungen/Ergebnisbericht-PLA-Abfaelle.pdf (accessed 12 June 2019).

[bibr10-0734242X211003969] Interessengemeinschaft der Thermischen Abfallbehandlungsanlagen in Deutschland (2015) Analyse/Beschreibung der derzeitigen Situation der stofflichen und energetischen Verwertung von Kunststoffabfällen in Deutschland [Analysis/description of the current situation of material and energetic recycling of plastic waste in Germany]. Available at: https://www.itad.de/information/studien/ITADConsulticKunststoffstudieApril2015.pdf (accessed 21 January 2020). [In German.]

[bibr11-0734242X211003969] Jambeck J Geyer G Wilcox C, et al. (2015) Plastic waste inputs from land into the ocean. Science 347: 768–771. 25678662 10.1126/science.1260352

[bibr12-0734242X211003969] Masoumi H Seyed Mohsen Safavi SM Khani Z (2012) Identification and classification of plastic resins using near infrared reflectance spectroscopy. International Journal of Mechanical and Industrial Engineering 6: 213–220.

[bibr13-0734242X211003969] Niaounakis M (2013) 5 - Physical recycling. In: Niaounakis M (ed.) Biopolymers Reuse, Recycling, and Disposal: Plastics Design Library. Oxford: William Andrew Publishing, 151–166.

[bibr14-0734242X211003969] Pedregosa F Varoquaux G Gramfort A, et al. (2011) Scikit-learn: Machine learning in python. Journal of Machine Learning Research 12: 2825–2830.

[bibr15-0734242X211003969] Piemonte V Gironi F (2013) Kinetics of hydrolytic degradation of PLA. Journal of Polymers and the Environment 21: 313–318

[bibr16-0734242X211003969] Recyclingmagazin (2017) RE|PLA Cycle bietet Lösungen für PLA-Recycling. Available at: https://www.recyclingmagazin.de/2012/02/22/repla-cycle-bietet-loesungen-fuer-pla-recycling (accessed 26 January 2021).

[bibr17-0734242X211003969] Rujnić-Sokele M Pilipović A (2017) Challenges and opportunities of biodegradable plastics: A mini review. Waste Management & Research 35: 132–140. 28064843 10.1177/0734242X16683272

[bibr18-0734242X211003969] Scaffaro R Maio A Sutera F, et al. (2019) Degradation and recycling of films based on biodegradable polymers: A short review. Polymers 11: 651. 10.3390/polym11040651 PMC6523205 30970659

[bibr19-0734242X211003969] Shen L Haufe J Patel M (2009) Product Overview and Market Projection of Emerging Bio-Based Plastics. Copernicus Institute for Sustainable Development and Innovation. Available at: http://news.bio-based.eu/media/news-images/20091108-02/Product_overview_and_market_projection_of_emerging_bio-based_plastics,_PRO-BIP_2009.pdf (accessed 26 December 2019).

